# Association of Increased Levels of lncRNA H19 in PBMCs with Risk of Coronary Artery Disease

**DOI:** 10.22074/cellj.2019.5544

**Published:** 2018-08-07

**Authors:** Sara Bitarafan, Mohsen Yari, Mohammad Ali Broumand, Sayyed Mohammad Hossein Ghaderian, Mahnoosh Rahimi, Reza Mirfakhraie, Faezeh Azizi, Mir Davood Omrani

**Affiliations:** 1 Department of Medical Genetics, Faculty of Medicine, Shahid Beheshti University of Medical Sciences, Tehran, Iran; 2 Department of Molecular Pathology, Tehran Heart Center Tehran University of Medical Sciences, Tehran, Iran; 3 Department of Bioinformatics and Genomics, Pharmacogenetic Research Center, Simple LIMS, San Diego, CA, USA

**Keywords:** Atherosclerosis, Coronary Artery Disease, H19, Long Non-Coding RNA

## Abstract

**Objective:**

Considerable research shows that long non-coding RNAs, those longer than 200 nucleotides, are involved in several human diseases such as various cancers and cardiovascular diseases. Their significant role in regulating the function of endothelial cells, smooth muscle cells, macrophages, vascular inflammation, and metabolism indicates the possible effects of lncRNAs on the progression of atherosclerosis which is the most common underlying pathological process responsible for coronary artery disease (CAD). The aim of present study was to assess whether the expression of the lnc RNA H19 was associated with a susceptibility to CAD by evaluating the expression level of H19 in the peripheral blood.

**Materials and Methods:**

A case-control study of 50 CAD patients and 50 age and sex-matched healthy controls was undertaken to investigate whether the H19 lncRNA expression level is associated with a CAD using Taqman Real-Time polymerase chain reaction (PCR).

**Results:**

The subsequent result indicated that the H19 lncRNA was over-expressed in CAD patients in comparison with the controls. However, it was not statistically significant. This overexpression may be involved in coronary artery disease progression.

**Conclusion:**

We report here, the up-regulation of H19 lncRNA in the whole blood of CAD patients and suggest a possible role for H19 in the atherosclerosis process and its consideration as novel biomarker for CAD.

## Introduction

One well-cited assertion in the literature is that coronary artery disease, described as a remodeling and narrowing of the coronary arteries which supply the required oxygen to the heart, is an important medical and public health issue and is also the leading cause of mortality worldwide (1, 2). Evidence from the Global Burden of Disease study shows that coronary artery disease (CAD) accounts for the biggest section of Disability-adjusted life years (DALYs) in 2010. It has been well established that atherosclerosis, the most common underlying pathological process responsible for CAD is a silent progressive chronic process described by the accumulation of cells, connective-tissue elements, lipids, fibrous elements, and inflammatory molecules in the vessel walls (1, 3). It is well known that CAD has a multifactorial origin related to environmental and genetic factors which lead to the disease both individually and through their interaction. Several studies have identified many risk factors for CAD such as hypertension, diabetes mellitus, dyslipidemia, obesity, birth weight, smoking, plasma homocysteine, physical activity, gender, and genetic variance. Conventional risk factors, such as hypertension, diabetes mellitus, dyslipidemia, and obesity are also considered to have a multifactorial origin caused by the interplay between genetic and environmental factors, as in the case of a CAD. It is estimated that CAD has a heritability of 50 to 60% (1, 4).

There is well recognized that only about 1.5% of the human genome is an influential part of encoding proteins and 98% of the human genome encodes for non-coding transcripts (5, 6). Considerable research studies have shown that the lncRNAs, non-coding RNAs longer than 200 nucleotides, play a significant role in regulating the function of endothelial cells, smooth muscle cells, macrophages, vascular inflammation, and metabolism indicating the possible effects of lncRNAs in the progression of atherosclerosis.

Several writers have addressed the correlation between the altered expression level of lncRNAs and several diseases suchas age-related cardiovascular diseases. A number of studieshave also demonstrated the existence of ncRNAs in the bloodstream which propose the possibility of using ncRNAsas prognostic and diagnostic biomarkers by measuring theirexpression levels in blood samples (5).

Substantially worthy efforts are already being done to reveal the role of ANRIL, Antisense Non-coding RNA in the INK4 Locus, in CAD. Furthermore, several studies show that H19, a well-known paternally imprinted and maternally expressed gene that is located close to the telomeric region on chromosome 11 in human (11p15.5), produces a 2.3 kb spliced, capped and polyadenylated lncRNA (6-10). The expression of H19 is upregulated during embryogenesis and dramatically down-regulated shortly after birth (6). Although the H19 lncRNA is undetectable in normal arteries, it is highly expressed in neointima after injuries and human atherosclerotic lesions. This expression can imply that the vascular response to injury is amalgamated by the re-expression of several fetal gene networks (11, 12). 

According to Ballantyne et al. (12), the first exon of H19 encodes miR-675 which is known to act as an effective biomarker in heart failure patients. Kallen et al. (8) found that H19 can bind both canonical and non-canonical binding sites of the let-7 miRNAs and modulates let-7 availability through acting as a molecular sponge that leads to reduced levels of free let-7 capable of binding to its target mRNA. Since the defective function of let7 is related to different types of cardiovascular disease, H19 can have a significant effect on CAD pathogenesis. Gao et al. (10), in a study conducted within a Chinese population, examined H19 polymorphisms and found them to be associated with the risk and severity of coronary artery disease. Zhang et al. (13) measured the circulating levels H19 in the plasma of 300 CAD patients and found significantly increased levels of H19 in patients with CAD. To date, no data has been published on the association of H19 lncRNA expression levels in peripheral blood mononuclear cells (PBMC) and the risk of CAD. The present study was conducted to determine the correlation between H19 lncRNA expression levels and the risk of CAD susceptibility in Iranian patients compared to controls both of which were confirmed by a coronary angiography. 

## Materials and Methods

The present study was a case-controlled study of 50 CAD patients of Coronary Artery Disease, confirmed by coronary angiography, (15 females and 35 males, mean age: 53.8 ± 7.3) and 50 age and sex-matched healthy controls, who underwent coronary angiography but did not have coronary artery disease, (15 females and 35 males, mean age: 51.6 ± 8.8), all of whom were referred to the Tehran Heart Center, in Tehran, Iran in 2015. Patients with cancer, acute myocardial infarction, severe heart failure (left ventricular ejection fraction =30%), cardiomyopathy, active infection and connective tissue disease were excluded. The participant characteristics with respect to age, sex, smoking status, hypertension (HTN) status, diabetes mellitus (DM) status, etc. are summarized in Table 1. A 2cc peripheral blood sample was collected from each participant in both experimental and control groups, and RNA extraction was performed immediately after sampling. 

### RNA isolation and cDNA synthesis

The total RNA was extracted from the whole blood samples with QIAamp RNA Blood Mini Kit (Qiagen, Germany), according to the manufacturer’s instructions. The total RNA was eluted with RNase-free water and stored at -80°C. RNA concentration of all samples was determined by a Nano-drop spectrophotometer. cDNA was synthesized from 1 µg of total RNA using QuantiTect Reverse Transcription Kit (Qiagen, Germany), according to manufacturer’s instructions.

### Real time polymerase chain reaction 

Real-time polymerase chain reaction (PCR) was performed in triplicates using RealQ Plus 2x Master Mix for Probe without ROX™. Reactions were run on the Rotor-Gene Q (Qiagen, Germany). The *H19* forward primer (TGCTGCACTTTACAACCACTG) is located in the 4th exon of the human *H19* gene, and the reverse primer (ATGGTGTCTTTGATGTTGGGC) 5^th^ is located in the exon. The TaqMan probe (TCGGCTCTGGAAGGTGAAGCTAGAGGA) spans the junction of the fourth and fifth exons to avoid false- positive results caused by DNA contamination (14). Gene expression levels for each sample were normalized to the expression level of ß-actin as a housekeeping gene. ß-actin amplification was performed using specific primers (sense: 5´CCTGGCACCCAGCACAAT3´, antisense: 5´GCCGATCCACACGGAGTACTT3´and probe:5´AT CAAGATCATTGCTCCTCCTGAGCGCA3´). 

### Statistical analysis

The efficiency for each real-time PCR reaction was determined through the LinReg method, and the expression level of the H19 lncRNA gene was estimated using REST 2009 software. Statistical analysis was carried out with SPSS (SPSS 14.0, Inc, Chicago, IL, USA). A P<0.05 was considered significant. Normality of distribution was assessed using the Kolmogorov-Smirnov test. Receiver operating characteristic (ROC) curve was utilized to evaluate the sensitivity and specificity of H19 lncRNA as a diagnostic marker for detecting CAD.

### Coronary angiography

Quantitative appraisal of CAD was performedthrough coronary angiography. Briefly, significantCAD was characterized as the vicinity of luminal width narrowing of 50% in the left anterior descending artery, left circumflex vein, right coronary supply route and their primary branches. Left primary trunk stenosis was considered as two-vessel sickness. The seriousness of coronary atherosclerosis was further classified as 1-, 2- or =3-vessel ailment as per number of coronary vessels with critical stenosis.

### Ethical considerations

All the experiments were performed in accordance with relevant guidelines and regulations. All blood samples were obtained from the Tehran Heart Center. This study was approved by the Ethics Committee of Shahid Beheshti University of Medical Sciences, and Tehran Heart Center, affiliated to Tehran University of Medical Sciences, Tehran, Iran. Written informed consent was obtained from each participant before blood sample collection.

## Results

### Characteristics of study subjects

The clinical characteristics of individuals with and without coronary artery disease is summarized (Table 1). A total of 50 subjects with coronary artery disease (CAD) and 50 controls were enrolled in the study. Hypertension, diabetes mellitus, and dyslipidemia are well-known risk factors of CAD (15). In the present study, CAD patients had higher levels of HTN, DM, and dyslipidemia compared to the control group, although it was not significant. 

### The expression level of H19 is increased in coronary artery disease patients

The result of the present study indicated that the H19 lncRNA was over-expressed in CAD patients in comparison with the controls. The over-expression of the H19 lncRNA was associated with both male and female genders (expression ratio=1.544, P=0.584 and expression ratio=1.932, P=0.521) respectively. However, this overexpression was not statistically significant.

### The receiver operating characteristic curve analysis

**Fig.1:**
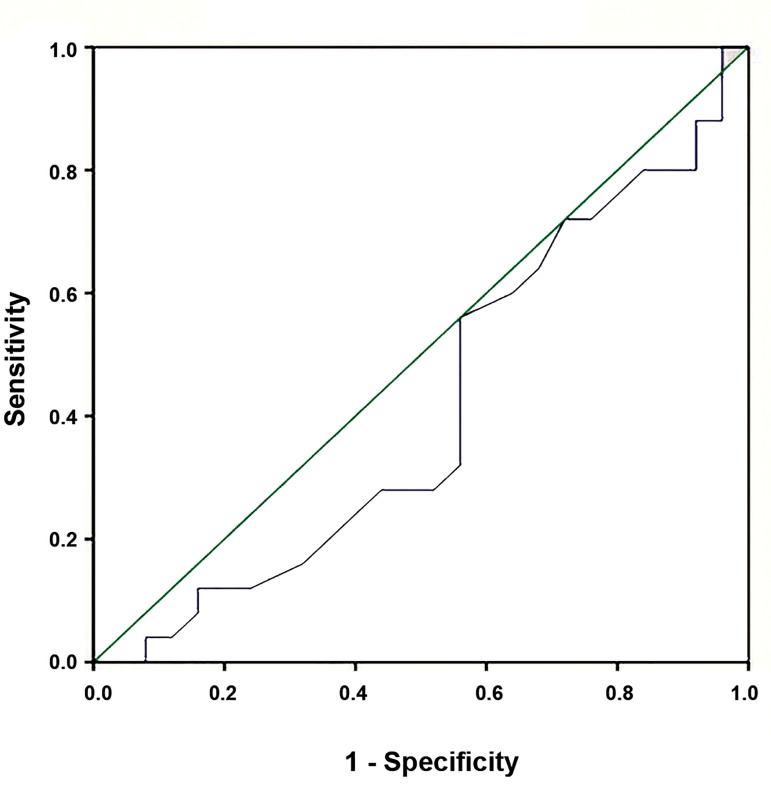
ROC curve of H19 expression measured by real-time PCR that could be used to predict the susceptibility to CAD in terms of sensitivity and specificity. ROC; Receiver operating characteristic, PCR; Polymerase chain reaction, and CAD; Coronary artery disease.

The ROC curve analysis was also indicated that the sensitivity of H19 for predicting CAD was 56%. The specificity of these transcripts was estimated at 44% (Fig .1).

**Table 1:** Clinical characteristics of individuals with and without coronary artery disease


Variable	Patient (n=50)Mean ± SD or n (%)	Control (n=50)Mean ± SD or n (%)	P value

Age (Y)	53.68	51.52	0.370
Female/Male	15 (30)/35 (70)	15 (30)/35 (70)	1
Smoking	8 (16)	9 (18)	0.500
Hypertension	25 (50)	21 (42)	0.274
Diabetes mellitus	15 (30)	10 (20)	0.178
LDL cholesterol (mg/dl)	123.39 ± 31.09	103.25 ± 40.6	0.990
HDL cholesterol (mg/dl)	37.57 ± 10.18	40.83 ± 10.63	0.811
TG (mg/dl)	152.96 ± 68.05	141.67 ± 78.81	0.602
TC (mg/dl)	167.26 ± 41.18	159.33 ± 43	0.522


LDL; Low-density lipoprotein, HDL; High-density lipoprotein, TG; Triglycerides, and TC; Total cholesterol.

## Discussion

Since the first clinical manifestation of CAD may lead to sudden death, primary prevention strategies in healthy individuals, who do not have any symptoms related to CAD, are essential. It is possible to classify individuals into different groups based on their estimated risk (low, intermediate, or high), and these classifications can be used to determine the intensity of preventive measures to be taken, from lifestyle recommendations to drug prescriptions. Several biomarkers, including genetic variants, have been investigated as candidates to improve risk estimation. However, little knowledge is available on the clinical utility of gene expression profiling in CAD patients which can improve the reclassification of individuals into more appropriate risk categories. For instance, cardiac-related or atherosclerosis-related lncRNAs with potential roles can be considered as novel biomarkers of CAD. 

The purpose of the present study was to investigate the correlation between the expression level of the H19 long non-coding RNA and coronary artery disease. According to the results of the study, the H19 long non-coding RNA was over-expressed in CAD patients in comparison to the controls. 

The exact mechanism underlying the overexpression of the H19 lncRNA in CAD patients is not well understood, but one possible explanation for this overexpression is the involvement of H19 in atherosclerosis (5, 16, 17), the main underlying pathological process responsible for coronary artery disease, and also in CAD risk factors such as hyperhomocysteinemia (16, 18, 19), HTN (20), diabetes mellitus (5, 7, 21, 22), and age (23). Like some lncRNAs, H19 has a relevant role in hypoxic endothelium and hence in the vascular physio-pathology even though the function of it is still largely unknown. One study has revealed that H19 inhibition results in decreased human umbilical vein endothelial cell (HUVEC) growth, inducing their arrest in the G1 phase of the cell cycle.

In addition to H19 which is known to be relevant to atherosclerosis (24), to the best of our knowledge, there are no previous reports about H19 lncRNA expression profiling in PBMC in human CAD patients to compare our results to and provide comparative conclusions directly. Recently Zhang et al. (13) from China measured the circulating levels of H19 lncRNA in plasma samples from 300 patients with CAD using qRT-PCR. They concluded that the plasma levels of H19 was significantly increased in patients with CAD which is consistent with the findings of the current article. They also suggested that increased plasma levels of H19 is associated with increased risk of CAD and H19 may be considered as a novel biomarker for CAD. However, in another study, it was shown that H19, which has no expression in adult smooth muscle cells, is re-expressed in altered atherosclerotic plaque cells. 

Gao et al. (10) investigated H19 polymorphisms and found that individuals with H19 risk alleles have a higher risk of CAD. These H19 lncRNA polymorphisms can cause more susceptibility to CAD. Since the H19 lncRNA has a high expression level during embryogenesis and is dramatically down-regulated after birth, the re-expression of the H19 in CAD patients may suggest that H19 has a significant role in the CAD pathogenesis process.

## Conclusion

The level of LncRNA H19 was examined in CAD patients, and increased level of H19 is associated with CAD. The use of gene expression profiling will improve the classification of individuals into more appropriate risk categories. According to our data, risk prediction can be optimized using a combination of prognostic molecular markers, including gene expression-based predictors. Overall, using lncRNAs as a biomarker has a plethora of benefits especially when it is possible to detect them easily in biological fluids. In addition to using lncRNAs as prognostic and diagnostic biomarkers, several companies are developing ncRNA-based strategies for diagnosing different types of diseases such as Cardiovascular Disease (CVD). Therefore, the H19 lncRNA may be used as a predictive biomarker for CAD, but the significance of H19 lncRNA expression levels needs to be investigated more thoroughly in further studies using a larger sample in order to be considered as a novel biomarker for CAD. 
